# Strategies for the multiplex mapping of genes to traits

**DOI:** 10.1186/1475-2859-12-99

**Published:** 2013-10-30

**Authors:** Andrew Garst, Michael Lynch, Ron Evans, Ryan T Gill

**Affiliations:** 1 Department of Chemical and Biological Engineering, University of Colorado, Campus Box 592, Boulder, CO 80303, USA; 2 Biomedical Engineering, Pratt School of Engineering, Duke University, 136 Hudson Hall, Campus Box 90281, Durham, NC 27708, USA; 3 OPX Biotechnologies, Inc., 2425 55th Street, Suite 100, Boulder, CO 80301, USA

**Keywords:** Genomic vector libraries, Multiplexed recombineering, Transposon saturation mutagenesis, Whole-genome sequencing

## Abstract

Rewiring and optimization of metabolic networks to enable the production of commercially valuable chemicals is a central goal of metabolic engineering. This prospect is challenged by the complexity of metabolic networks, lack of *complete* knowledge of gene function(s), and the vast combinatorial genotype space that is available for exploration and optimization. Various approaches have thus been developed to aid in the efficient identification of genes that contribute to a variety of different phenotypes, allowing more rapid design and engineering of traits desired for industrial applications. This review will highlight recent technologies that have enhanced capabilities to map genotype-phenotype relationships on a genome wide scale and emphasize how such approaches enable more efficient design and engineering of complex phenotypes.

## Introduction

Optimizing microbial metabolism for the production of commercially valuable chemicals such as biofuels, chemicals and therapeutics is a central aim of metabolic engineering. This aim is typically approached by altering native metabolic networks to promote flux through desired metabolic pathways while minimizing the buildup of potentially toxic intermediates and the formation of undesired byproducts. Towards this end, a variety of rational engineering approaches have been successfully applied. For example, the introduction of non-native pathways to promote product formation [1], the over-expression of native biosynthetic enzymes [2], the removal of regulatory repression [3], and modifications made to increase precursor metabolite supply [4] have all proven effective for improving product yields. Such rational modifications however require significant *a priori* knowledge of the pathways in question [5,6]. In many cases this knowledge is incomplete, particularly for complex phenotypes that require an intricate balance between the activities of many seemingly unrelated gene products.

**Figure 1:**
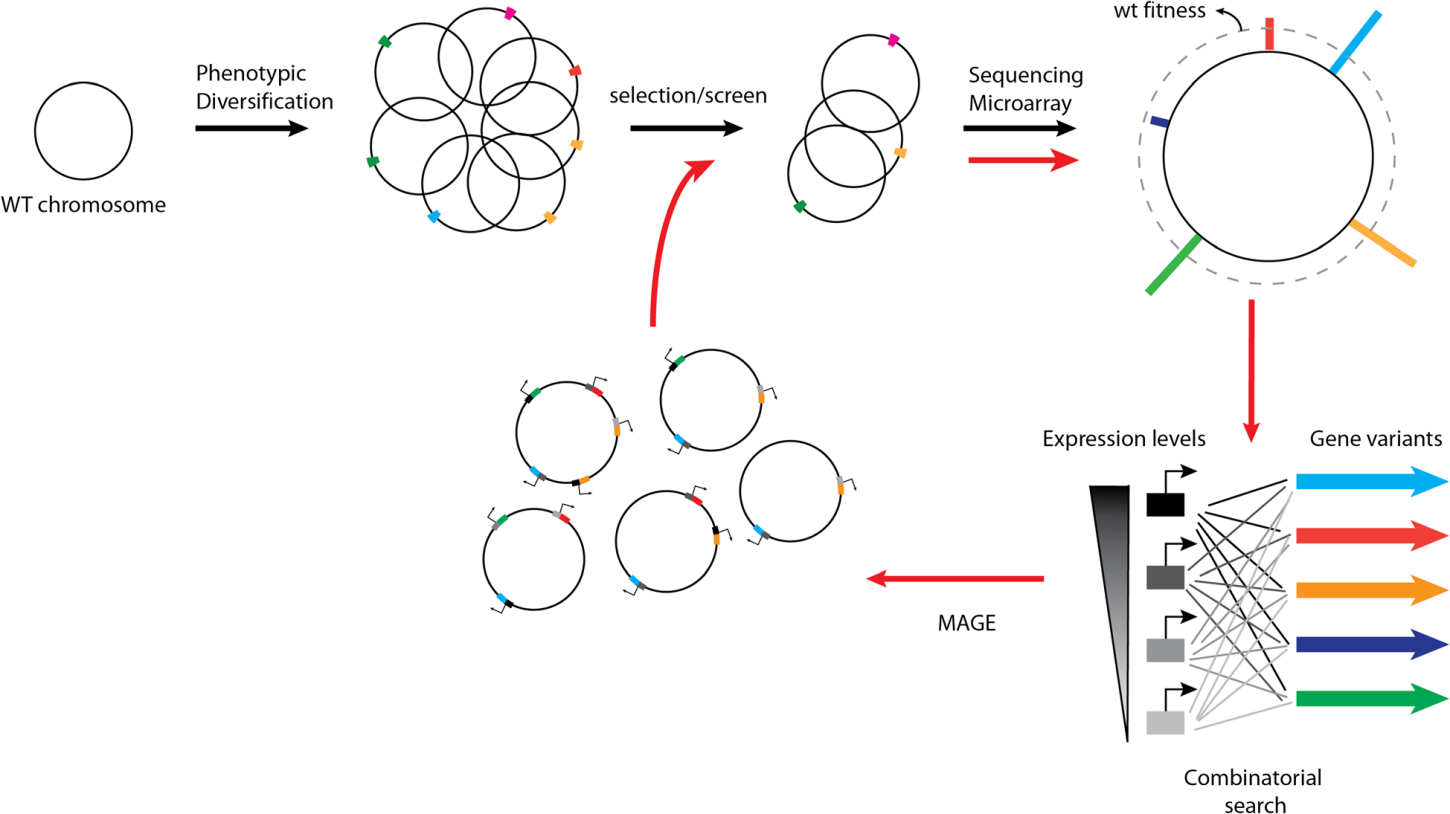
**Overview of inverse engineering approaches.** Inverse engineering begins with a wild-type cell line in which a large number of mutations are introduced in either a random or directed manner to generate phenotypic diversity. These mutants are subjected to screening or selection for a trait of interest (i.e. solvent/temperature tolerance or metabolite production). The pre and post selection populations are sequenced or genotyped by microarray hybridization. The fitness (*W*_*i*_) of the each genotype (*g*_*i*_) can be computed by the equation to obtain a plot of fitness (far right circle) where each bar represents a specific genetic loci. Alleles identified by this can be used to guide more focused combinatorial optimization on the trait of interest using directed techniques such as MAGE [16]. While this provides a promising algorithm for directed evolution on the genome scale future technologies will need to be developed to enable this engineering cycle to be preformed rapidly and recursively (indicated by the red arrows) by tracking gene combinations in a high-throughput fashion.

In contrast to rational engineering approaches, “inverse” metabolic engineering approaches employ directed evolution to rapidly explore large adaptive landscapes in search of beneficial mutations [7,8]. Traditional genome engineering methods such as chemical mutagenesis or genome shuffling [9] however generate mutations in a random and combinatorial fashion and require extensive sequencing and characterization to assess genotype-phenotype correlations and distinguish between adaptive mutations and neutral or maladaptive hitch-hiking mutations [10,11]. The immense combinatorial sequence space of even a modest genome size (~4^4,000,000^) for the *Escherichia coli* genome) requires more rational search strategies that can identify genes or gene networks that promote the desired phenotype in laboratory timescales (Figure 1). Such information, can then be leveraged to guide an exploitative combinatorial optimization of the most relevant genes [12], akin to the use of structural and evolutionary information to guide site saturation mutagenesis for protein engineering [13]. The development of platform technologies that enable genome wide mapping of genes to traits has thus been a central challenge for the development of more efficient strain engineering.

A variety of techniques have been recently developed to address these challenges and enable targeted approaches to genome wide modification and tracking of genotype fitness that are ultimately aimed at speeding up the genome engineering cycle (Figure 1) [7,8,14]. These multiplex “forward” genomics approaches are founded on fundamental technological advances in both DNA sequencing and microarray based DNA detection methods that allow quantitative tracking of the concentration of different genotypes in a large population [8,12,14]. Additionally, recent advances in multiplexed DNA synthesis [15] and the ability to rationally modify bacterial and eukaryotic chromosomes using homologous recombination have enabled the production of genome scale libraries with characteristics that are more suitable for the short read sequencing and array based detection [16-21]. This review will focus on approaches to genome wide mapping of genotype-phenotype relationships and discuss how such methods are being applied to advance a basic understanding of and ability to design and engineer complex phenotypes.

### Genomic vector library enrichment strategies

**Figure 2:**
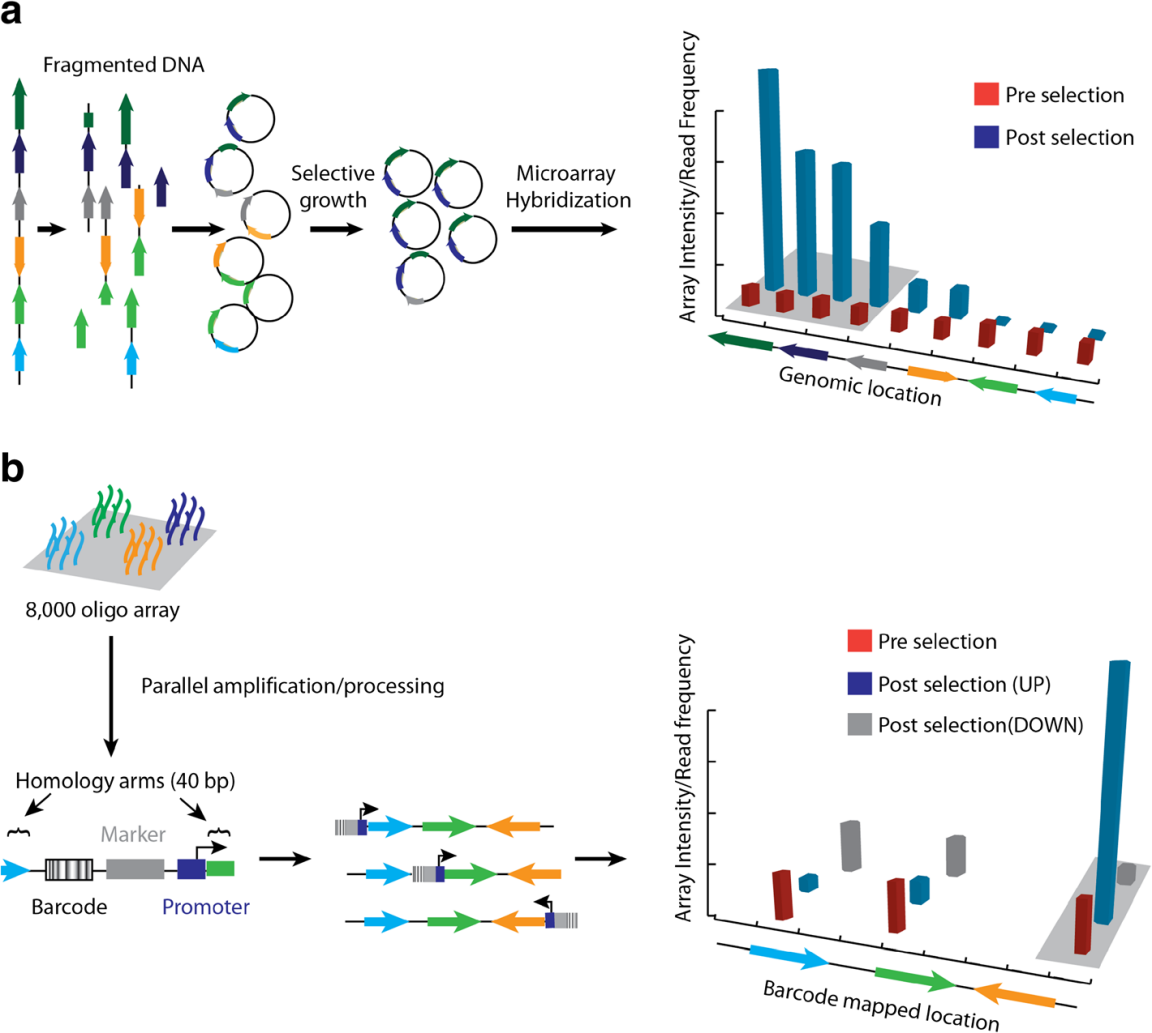
**Genome wide fitness measurement techniques. a)** Vector library based approaches such as SCALEs [25] start with cloning of random DNA fragments that represent different sites of the genome of interest. This diverse vector library is transformed into the host strain and grown under a defined selective pressure. Following selection the cells are lysed and the vector libraries cut and hybridized to a microarray. The signal intensities from the microarray are then mapped back to the genomic coordinates to identify traits of interest and measure fitness as described in Figure 1. **b)** Overview of TRMR. An array of rationally designed oligos are cleaved and processed in parallel to produce cassettes with homology arms that guide rational engineering of the entire genome. The TRMR barcodes can then be hybridized to a microarray or PCR amplified and sequenced to determine the fitness of each mutation. This technique provides a versatile method for engineering different functional changes into the genome of interest and rapidly tracking the effects.

One of the most well established methods for mapping genes to fitness at the whole genome scale involves the creation of extra-chromosomal libraries of fragmented genomic DNA. In these approaches purified genomic DNA is digested and cloned into a plasmid backbone and transformed into a suitable host strain (Figure 2a). Following application of selective pressure or a high-throughput screen, vectors containing the enriched genomic fragments are isolated and subsequently identified by hybridization to whole genome microarrays or by next-generation sequencing [7,22-24] (Figure 2a). This strategy was first demonstrated using high-density whole genome microarrays to identify protein- protein interactions implicated in mRNA splicing using a yeast two-hybrid screen [22]. Interactions were identified by co-expression of a DNA binding fusion protein of interest with an genomic activator fusion library and allowed high-throughput array based detection of interacting library variants from pooled clones [22]. Subsequent studies using genomic libraries in *E. coli* demonstrated that this approach could be implemented to identify genes that confer tolerance to the antimicrobial agents in Pinesol [23].

Genomic vector libraries approaches have recently been used to enable more sophisticated genotype-phenotype mapping. For example, MultiSCalar Analysis of Library Enrichments (SCALEs) allows simultaneous investigation of the fitness conferred by individual genes, multi-gene fragments, and small operons [25]. SCALEs has been successfully applied to the identification of genes that improve growth under antimetabolite stress [26], genes that restore redox balance in *E.coli* strains evolved for succinate production [27], genes that confer tolerance to a variety growth inhibiting compounds relevant to cellulosic biofuels [28-31], as well as to investigate the basic mechanisms at work in laboratory growth selections [32]. For example, it was demonstrated that single batch growth predominantly favors microbes with increased growth rate while serial batch culturing provides selective pressure for both increased growth rate and decreased lag time. These results agreed well with a simple mathematical model of bacterial growth, suggesting a growing importance for mathematical modeling as multiplex fitness mapping technologies continue to develop [32].

One potential limitation of many vector based libraries is the inability to identify phenotypes that arise from synergistic interactions between distantly spaced loci. A recently described technique known as Coexpressing Genomic Libraries (CoGEL) was developed to overcome this limitation by constructing genomic libraries in multiple vectors with different replication origins and resistance makers that can be co- expressed in individual cells [33]. This approach was demonstrated to successfully rescue the auxotrophy of a designed mutant strain in which two mutations were introduced into the lysine biosynthetic pathway at distal chromosomal loci. To demonstrate the utility of this approach for the study of more complex phenotypes, CoGel variants were isolated from *E. coli* exposed to acid stress [33]. In addition to recovering genes involved in known proton exchange pathways the study identified unanticipated roles for a small RNA (*arcZ*) and *recA* in acid tolerance [33].

A major challenge for these approaches, as well as sequencing in general, involves the parallel sequencing of multiple sites across individual genomes within a complex cellular mixture. That is, while it is now possible to sequence multiple sites across a population in parallel, methods for determining which mutations came from the same individual cell remains difficult.

### Identification of novel functional activities from metagenomic vector libraries

Natural microbial communities provide a rich source of diversity that can be prospected for novel metabolic activities and used to expand the capabilities of genetically tractable organisms such as *E. coli*. This approach is founded on techniques that were first developed to enable extraction and direct cloning of environmental DNA to perform 16s based rRNA profiling for microbial ecology studies [34]. These techniques have since been expanded upon for the purpose of identifying novel catalysts that perform industrially relevant reactions [35,36]. For example, shotgun based cloning of metagenomic DNA and activity screening approaches have been used to identify novel amylases [37] and cellulases with enhanced stability and activity compared to the enzymes represented in cultured organisms [38]. These enzymes represent important industrial biocatalysts as their activities are critical to the utilization of cellulose for the production of next-generation biofuels.

The complexity of metagenomic samples can limit the efficiency with which novel activities can be successfully identified, thus necessitating strategies to enrich the functional traits of interest prior to cloning. For example, exposing natural microbial communities to a selective pressure of industrial interest (i.e. thermotolerance, carbon substrate utilization, etc.) has proven effective for enriching the metabolic functions that have evolved under similar conditions in nature. This approach was elegantly demonstrated in a study that identified genes associated with carbon uptake in sediments taken from Lake Washington [39]. Cultures were grown in the presence of single carbon C^13^-labeled substrates to enrich the organisms responsible for carbon uptake and their genomic DNA isolated by density gradient centrifugation [39]. Target gene enrichment has also been achieved using degenerate PCR primers to amplify the gene families or pathways of interest using phylogenetically conserved priming sites [40]. Homologous recombination based cloning using the RecET system in *Escherichia coli* has also recently proven useful for bioprospecting large heterologous gene clusters that perform coordinated biochemical functions [41]. This technique has enabled direct cloning and characterization of polyketide synthase gene clusters ranging from ~10-50 kb from a number of organisms [41]. These gene clusters are of keen interest as they are responsible for the production of many clinically important secondary metabolites [42,43].

### Transposon saturation mutagenesis

Disruption or modification of genes within the genome provides another useful method for rapid and parallel dissection of gene functions. Transposon saturation mutagenesis has been widely employed due to the efficacy of transfection and the wide variety of bacterial species for which this technology exists [44-49]. Transposon libraries are constructed using modified transposons that enable downstream identification of the genomic insertion sites by microarray or sequencing. For example, one recently developed strategy name Tn-Seq takes advantage of the type-IIS MmeI enzyme to cleave 20 nucleotides outside of its recognition site to identify adjacent genomic sequences [45]. Transposon mutagenesis approaches have been employed to study many aspects of adaptive bacterial physiology including gene fitness under different media conditions [44], bacterial pathogenesis [46,50], biofilm formation [51], and motility [47]. The high efficiency with which these libraries can be constructed has also enabled their use in examining pairwise interactions through combinatorial gene knockout strategies [45].

In addition to studying the genetics of naturally occurring phenotypes, transposon libraries have also been utilized to study traits evolved by directed evolution for the purposes of industrial applications. For example, a recent study combined transposon and plasmid overexpression libraries to investigate the molecular mechanisms of ethanol tolerance in *E. coli*[52]. The study identified key modules involved in maintaining membrane and cell wall integrity, as well as transcription factors that regulate osmolyte production and ethanol degradation [52]. Similarly, Alper *et. al.* have utilized transposon library approaches to identify genes that increase lycopene production titers in *E. coli*[53,54]. These studies collectively identified a number of genes that boost lycopene production but were not identified by stoichiometric modeling based approaches, thus demonstrating the complementarity of inverse and rational metabolic engineering approaches.

### Rational genomic libraries using homologous recombination

Despite the widespread use of transposon- or plasmid-based libraries, the data generated can be difficult to interpret, low-resolution, and non- or semi-quantitative. Moreover the types of mutations introduced are random and typically limited to increased dosage or insertions/disruptions. As an example, many insertions can result in partial disruption of transcription or translation of the gene target, making it difficult to account for the functional influence of the mutation. As a second example, more sophisticated genome-engineering approaches require capabilities for modifying specific regions of the genome at a resolution as fine as the single nucleotide level. Homologous recombination is well suited to this task as it allows precise genomic manipulations across the entire genome [55-57]. The utility of homologous recombination for directed genome engineering was first demonstrated with targeted single gene replacements in *Saccharomyces cerevisiae*[58]*.* Subsequently it was demonstrated that this strategy could be expanded to enable rapid parallel identification of gene knockouts by inclusion of a 20 base pair DNA tag in the replacement cassette that can serve as a barcode for microarray based quantification of each allele knockout [19]. Homologous recombination has since been applied at a genome wide scale to enable parallel assessment of allele fitness during growth in a variety of conditions [20] and more recently to study filamentous growth in yeast as this phenotype is characteristic of opportunistic yeast pathogens [59]. Similarly in *E. coli* it has been demonstrated that endogenous *recET* or λ-red bacteriophage genes can mediate highly efficient recombination in bacteria [17,55,56]. This has enabled the construction of similar genome wide libraries in *E. coli* that serve as a valuable tool for the research community [57].

More recently, recombineering has been demonstrated to provide an important tool for identification of genes that confer increased fitness under conditions that are common to industrial settings. For example, Warner *et al.* reported the trackable multiplex recombineering (TRMR) [21] technique, which combines the barcoding strategy developed in yeast [19] with the power of homologous recombination to efficiently engineer genome wide libraries in a single transformation (Figure 2b). The authors demonstrated that such a library can be accomplished on a ~1 week timescale using state of the art DNA microarray fabrication technologies that enable parallel synthesis of a large number of defined oligonucleotides [15,60-62]. TRMR has been employed to identify alleles that improve fitness in the presence of alternative carbon sources, antimetabolites and cellulosic hydrosylate as these represent commonly encountered stresses that bacteria are faced with in industrial applications [21]. Interestingly TRMR established a previously unidentified role for *aphC* in conferring tolerance to cellulosic hydrosylate. The ability to generate, select, and genotype TRMR libraries on a rapid time scale (~ 1-2 weeks) significantly enhances the throughput of target identification, and the design of similar genome wide cassettes employing a broader range of mutations is envisioned. Additionally, employing TRMR recursively to generate combinatorial genome wide mutations could potentially enable in depth analysis of genetic interactions important for these and other phenotypes.

Another application of recombineering has been the creation of combinatorial genetic libraries that are superior in many ways to those generated by random mutation techniques. For example, Multiplex Automated Genome Engineering (MAGE) takes advantage of the high efficiency (~3-30%) of ssDNA mediated allelic replacement to introduce defined mutations at multiple chromosomal loci [16] (Figure 1). To demonstrate the power of MAGE, Wang et al. chose 24 genes that had been previously identified to enhance lycopene production and combinatorially optimized their expression using oligonucleotides that introduce degeneracy into the ribosome binding site (RBS) of these genes [16]. This approach generated ~ 10^8^ mutations/day and ultimately allowed for the identification of strains within 3 days that had ~ 5 fold increased lycopene production. Similarly, MAGE was utilized for combinatorial introduction of T7 promoters upstream of genes involved in aromatic amino acid production in *E. coli* to rapidly survey how these expression changes influence pathway flux using a colorimetric assay for indigo production [63]. MAGE has been also been further extended to enable rapid heiarchical assembly of mutations across the entire genome via conjugation [64]. This approach known as conjugative assembly based genome engineering (CAGE) provides a viable alternative to synthetic genome assembly techniques [65,66] for rapidly constructing chromsomes with a large number of mutations (~10^2^/genome).

The combination of TRMR and MAGE to globally search and locally optimize metabolic networks respectively offers an exciting algorithm for approaching metabolic engineering of complex traits (Figure 1) [12]. Sandoval *et al*. reported a first attempt to combine these methodologies for the purposes of optimizing growth under industrially relevant conditions [67]. The authors selected genes identified by the TRMR data as adaptive for growth in acetate, low pH or cellulosic hydrosylate as targets for recursive RBS engineering by MAGE. Interestingly, many of the clones identified following recursive MAGE were identical to the single mutants identified by TRMR, suggesting the possibility of negative epistasis between the targeted alleles [67]. It is important to note however, that this strategy required isolation of individual clones and sequencing of each targeted locus, thus limiting the ability to deeply characterize the individual fitness profiles within the combinatorial library. The development of techniques that enable more rapid characterization of the fitness of such combinatorial genotypes from a mixed population thus represents an important challenge to the future of rational combinatorial genome-engineering.

### Expression profiling methods

**Figure 3:**
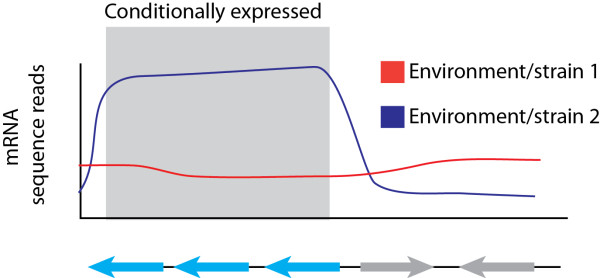
**Expression profiling to identify adaptive gene expression responses.** Changes in gene expression (red line vs. blue) are often correlated with adaptation to changes in the environment or genotypic alterations. Genome wide profiling of such expression changes can thus enable correlations or causal associations to be imputed between the affected genes and the trait of interest. Transcriptome analyses can also define transcriptional units (blue genes) that are co-expressed under varying conditions to allow annotations of unknown gene functions or associations between co-expressed genes.

Microorganisms have evolved exquisite transcriptional regulatory networks that enable rapid changes in gene expression and allow them to adjust their metabolism for optimal performance under a wide variety of environmental conditions. The ability to track genome wide changes in mRNA expression can thus provide another useful metric for identifying functional elements that are involved in various adaptive processes. In addition, transcriptome level analyses can help define the basic organization of operons and identify non-coding regulatory networks that shape cellular metabolism [68] (Figure 3). Unlike the genotyping methods described above, transcriptional profiling does not provide information on the relative fitness of differentially transcribed loci. It can however be utilized to survey genome wide changes in expression that are correlated with an observable phenotypic change [69-71], or gain insight into the rewiring of metabolic networks in evolved lineages [72]. Traditional transcription profiling methods have relied on tiling array [7,73] however advancements in DNA sequencing have enabled the development of direct whole cell RNA sequencing, or “RNA-seq” that allow a much greater dynamic range of detection than was previously possible [74]. Additionally, RNA-seq methods offer potentially unbiased approaches to analyze transcript levels and the strand origin of coding and non-coding messenger RNAs by avoiding cDNA synthesis and amplification steps [74], thus enabling more detailed understanding of the transcriptional landscape.

Transcriptome wide analysis has provided a number of recent insights into the structure of native transcription networks. For example, integration of transcriptional profiling with chromatin immunoprecipitation (CHIP) RNA polymerase II to identify global occupancy of the transcription machinery enabled the identification of a number of 4661 transcription units in *E. coli* as well as defining dynamic expression patterns for many operons [68]. In the case of the *thrLABC* operon for example, transcription was found to initiate from the most upstream promoter during log phase, but during lag phase an alternative promoter is activated to enable expression of the distal genes *thrB* and *thrC* and alleviate attenuation caused by the 5′ untranslated region in this polycistronic mRNA [68]. Such approaches have also yielded a wealth of information about the global rewiring of these networks during adaptation to different growth conditions. For example transcriptome wide analyses of *Bacillus subtilis* under 104 different growth conditions allowed for comprehensive identification of regulons associated with the various transcription factors in this industrially relevant organism [70].

Application of comparative transcriptome profiling between parental and evolved or engineered cell lines can also provide useful insight into the genes whose altered expression contributes to production and tolerance related phenotypes. For example, one study of ethanol tolerance in *E. coli* compared the transcription profile of a parental strain to multiple laboratory evolved strains and subsequently confirmed causal link between up-regulation of iron transport and amino acid biosynthesis genes [75]. Supplementation of these metabolites into a 5% ethanol containing media enhanced specific growth rates of the wild-type strain supporting this hypothesis. Another study looked at the difference between the transcriptome of a strain of a *E. coli* that was rationally engineered for valine production compared to the parental W3110-strain derivative [72]. The transcriptome of the valine producing strain exhibited increased levels of expression for the valine biosynthesis genes as intended and decreased expression of the tricarboxylic acid cycle enzymes, whereas carbon flux through glycolysis and the pentose phosphate pathway were unchanged [72].

In addition to monitoring changes in expression due to environmental perturbations transcriptional profiling has been recently employed to better understand transcriptional networks that have been rewired by synthetic means. For example, global transcription machinery engineering (gTME) involves the creation of plasmid encoded libraries of major sigma factors that have altered DNA binding preferences [76]. Following application of a selective pressure the mutant sigma-factors and the corresponding transcriptional changes can be characterized to identify processes that enhance survival or growth. Although gTME has been found to produce a large number of differentially expressed genes in evolved lineages [76,77], transcriptional profiling experiments have aided in the identification of genes that more than double the production of the essential amino acid L-tyrosine [78].

### Whole genome sequencing (WGS)

Despite the power to map genotype fitness genome wide, the techniques described above lack the ability to completely define the genotype of an engineered or evolved strain, leaving open the possibility that off target mutations may influence the observed trait. While whole genome sequencing (WGS) can address this challenge, it has historically been costly and labor intensive. However, advances in next generation sequencing have occurred at an astounding rate. Current technologies enable ~ 10^14^ kbp per sequencing run [79], and the increased length of real time sequencing technologies [80-82] promise to boost this output further.

Long term laboratory evolution studies now utilize WGS to map mutations onto growth phenotypes. WGS has therefore enabled exciting new insights into the molecular mechanisms of adaptation to a variety of stresses. For example, a recent study sequenced the genomes of 29 *E. coli* clones taken from different time points during the long term evolution of the population for increased citrate utilization [83]. The sequences revealed a gene duplication event that placed a non- transcribed citrate transporter gene *citT* in front of an actively transcribed promoter to enable gene expression. Another study recently performed 115 parallel selections for thermotolerance (growth at 42.2°C) followed by WGS of a single isolate from each population [84]. The study found that although adaptive mutations often do not precisely overlap at the nucleotide level, convergence can readily be detected at the single gene and operon level with relatively few isolates. The study also found epistatic “blocks” that seemed to provide different potential routes for further adaptation [84]. Notably, mutations identified using WGS included not only point mutations, but a number of gene duplications, insertions, deletions, and rearrangements all in combinatorial sets that would be difficult to identify by current tracking technologies.

On the other hand, although WGS provides a complete description of the genome, it is limited to relatively small sample sizes making it impossible to determine the relative fitness of different mutational combinations. To determine the fitness of combinatorial mutations requires deep sampling of the combinatorial sequence space of a directed set of mutations. As tracking technologies are expanded from single allele approaches to determining the fitness of many genetic combinations at high depth they will open the door to the evaluation of increasingly complex genotype-phenotype relationships. WGS should therefore be considered as a complementary approach to current and future tracking technologies that will aid in improving the accuracy with which relevant mutations are identified.

## Conclusions

To the benefit of basic and applied research alike, genome wide tracking technologies have significantly enhanced the throughput with which genotype- phenotype relationships can be investigated. Approaches such as TRMR [21] and SCALEs [25] for example can produce high resolution fitness maps covering the entire *E. coli* genome and have aided in uncovering genes implicated in a variety of industrially relevant traits with rapid turnover times. Recent efforts have been made to combine TRMR with combinatorial engineering strategies similar to MAGE [16] promise to significantly increase throughput of strain engineering programs (Figure 1) [29]. This study however highlighted the importance of epistatic interactions as many of the colonies isolated after combinatorial engineering contained only singly mutated ribosome binding sites as well as the need for technologies to more deeply analyze populations that have been engineered by multiplexed approaches.

The techniques described here will need to be improved such that multigenic traits can be characterized in parallel and the engineering cycle can be performed recursively. Recent progress towards more effective combinatorial tracking has been made using synthetic RNA based regulatory devices that enable multiplexed, sequence-specific gene control from single plasmid [85]. Similar approaches could also be readily envisioned using the recently described CRISPR system in which nuclease inactive *cas9* was demonstrated to provide inducible transcription repression based solely on the sequence of a synthetic guide RNA [86]. Sequence specified combinatorial libraries that sample different genes at varying expression levels in these systems therefore offer an exciting opportunity to more quickly survey adaptive landscapes in search of more optimal engineering solutions. Combinatorial tracking approaches also offer the promise of new sources of epistasis in the complex genetic networks of living organisms. Techniques such as expression profiling and WGS will also continue to provide complementary tools that enhance our knowledge of complex phenotypes and, importantly, our ability to engineer new and useful traits.

## Competing interests

Gill, Lynch, and Evans declare financial interest in Opxbio.

## Authors’ contributions

AG prepared all of the illustrations and served as lead author of the manuscript. ML, RE and RG provided supervision and editing of the manuscript. All authors read and approved the final manuscript.
